# Risk Factors and Pathogenesis of HIV-Associated Neurocognitive Disorder: The Role of Host Genetics

**DOI:** 10.3390/ijms19113594

**Published:** 2018-11-14

**Authors:** Ian Simon Olivier, Ramón Cacabelos, Vinogran Naidoo

**Affiliations:** 1 Division of Physiological Sciences, Department of Human Biology, Faculty of Health Sciences, University of Cape Town, Observatory 7925, South Africa; iansimonolivier@gmail.com; 2 EuroEspes Biomedical Research Center, Institute of Medical Science and Genomic Medicine, 15165 Bergondo, Corunna, Spain; rcacabelos@euroespes.com

**Keywords:** HIV-associated neurocognitive disorders, HIV-associated dementia, HIV-encephalitis, neuroAIDS, host genetics, risk factors, HIV, cognitive impairment, polymorphisms

## Abstract

Neurocognitive impairments associated with human immunodeficiency virus (HIV) infection remain a considerable health issue for almost half the people living with HIV, despite progress in HIV treatment through combination antiretroviral therapy (cART). The pathogenesis and risk factors of HIV-associated neurocognitive disorder (HAND) are still incompletely understood. This is partly due to the complexity of HAND diagnostics, as phenotypes present with high variability and change over time. Our current understanding is that HIV enters the central nervous system (CNS) during infection, persisting and replicating in resident immune and supporting cells, with the subsequent host immune response and inflammation likely adding to the development of HAND. Differences in host (human) genetics determine, in part, the effectiveness of the immune response and other factors that increase the vulnerability to HAND. This review describes findings from studies investigating the role of human host genetics in the pathogenesis of HAND, including potential risk factors for developing HAND. The similarities and differences between HAND and Alzheimer’s disease are also discussed. While some specific variations in host genes regulating immune responses and neurotransmission have been associated with protection or risk of HAND development, the effects are generally small and findings poorly replicated. Nevertheless, a few specific gene variants appear to affect the risk for developing HAND and aid our understanding of HAND pathogenesis.

## 1. Introduction

The human immunodeficiency virus (HIV) epidemic is a huge public health issue worldwide, and is prominent in developing countries. According to UNAIDS (2018), approximately 36.9 million people were living with HIV in 2017. Eastern and southern Africa have the greatest proportion of cases by far, with an overall prevalence of 7.4% [1]. HIV is spread by bodily fluids (blood, semen, breast milk, rectal and vaginal fluids), with sexual transmission generally being the most common, although sharing of drug injection equipment is also a common mechanism. HIV is a retrovirus that causes progressive disease resulting in immune system destruction through CD4+ T-cell, macrophage and monocyte infection [2]. If left untreated, HIV infection ultimately leads to acquired immunodeficiency syndrome (AIDS), which is associated with characteristic opportunistic infections due to a weakened immune system. The advent of combination antiretroviral therapy (cART) has drastically decreased the rate of progression of AIDS and death, leading to a much improved quality of life and approximating a near-normal life expectancy. Treatment has now improved to such an extent that HIV is presently viewed as a chronic disease, rather than a terminal illness [3].

HIV infection, however, still presents with considerable neurological pathology in daily clinical settings, which can affect the everyday functioning of patients [2,3]. Fortunately, since the introduction of cART, the prevalence of more severe neurocognitive conditions caused by opportunistic infections and the virus itself, such as HIV dementia, have decreased profoundly. The characteristic “AIDS dementia complex” is now much rarer. Nonetheless, HIV causes a variety of milder, yet no less common forms of neurocognitive impairments (NCIs), collectively named HIV-associated neurocognitive disorders (HAND). The prevalence of HAND amongst HIV positive individuals in South Africa has been found to be between 23.5%, and is even higher in other settings and countries, with up to 60% presenting with HAND, depending on the setting [4,5]. HAND comprises several different types of cognitive impairments, predominantly presenting with impairments in attention, memory [6], processing speed and executive function [7]. Emotional manifestations, such as depression and apathy, and, to a lesser degree, motor deficits also occur [2,3]. Impairments of memory and executive functioning often affects workplace functioning and medication adherence, as does depression [3,6]. Importantly, failure of HIV therapy, which is a common problem in developing countries, is associated with higher risk of progression of neurocognitive impairments [3]. In fact, nearly 58% of the burden of dementia occurs in low- and middle-income countries [8]. Many of those affected do not receive the treatment they require and experience severe cognitive and psychiatric problems, thus decreasing the quality of life for those patients [7,8]. The prevalence of dementia in developing countries is increasing, and it is expected that within the next 20 years, these countries will have 71% of all people living with dementia [8]. This is in direct contrast to Western Europe and the United States, reporting a 22–40% decrease in prevalence [9], and an overall incidence of 9% [10], respectively, which is attributed to improved education, lifestyle and living conditions. In South Africa, which is grappling with the HIV/AIDS epidemic, there are about 187,000 documented people living with dementia [11], suggesting a significant problem for the African continent as a whole. In South Africa, and other African countries, inequalities in the distribution of health care resources remain. People from poor socio-economic backgrounds have limited access to health care, with the additional problem that proper medical treatment is hampered due to a lack of resources in the South African public health care system [12,13,14]. Most patients cannot afford to access private health care due to the cost of the required medications, medical consultations and evaluations [15]. With a vital need for better therapeutic avenues, therefore, it is important to find effective health care treatments against dementia-related neurodegeneration, which includes HAND. 

## 2. Classification and Diagnosis of Human Immunodeficiency Virus-Associated Neurocognitive Disorder (HAND) Phenotypes

**Figure 1:**
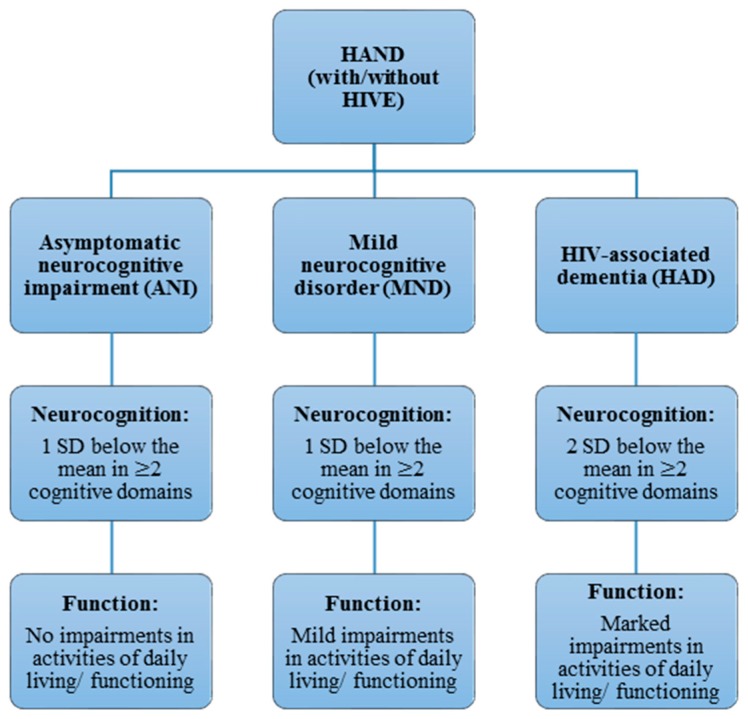
Categories and types of human immunodeficiency virus (HIV)-associated neurocognitive disorder (HAND), according to the Frascati criteria. When diagnosing patients with HAND, it is important to consider comorbidities, such as substance use and psychiatric illnesses, and to exclude other possible causes. Neurocognitive tests should evaluate at least the following: verbal/language skills, attention-information processing/working memory, memory (both learning and recall), motor skills, sensory perceptual, abstraction/executive function, and speed of information processing. Functional impairments, regarding mental acuity, social interactions, work, and other aspects, are usually self-reported, but witnesses are helpful [16,18]. HIVE, HIV encephalitis.

HAND is currently sub-classified into three forms, according to the Frascati criteria [16]: asymptomatic neurocognitive impairment (ANI), mild neurocognitive disorder (MND) and HIV-associated dementia (HAD) (Figure 1). This criteria has also been adapted by other countries on the African continent, such as South Africa [4]. All of those forms can present with or without HIV encephalitis (HIVE), the active infection of brain parenchyma by HIV, which has become less common in HAND with cART and is not a necessary feature of HAND pathogenesis [17]. HAD is the most severe, constituting a type of subcortical dementia, but has become relatively rare following cART [18]. HAD is diagnosed when at least two cognitive domains are impaired, and subtypes are differentiated by the presence and degree of impairment of everyday functioning [16]. As such, HAD and MND present with varying degrees of impairment in activities of daily living, while no such functional impairments are seen in ANI [16]. ANI is currently the most prevalent manifestation of HAND, including in developing countries like South Africa, while HAD is now relatively rare (less than 5%, depending on the setting) [5]. Importantly, the clinical usefulness or relevance of diagnosing the asymptomatic neurocognitive impairments remains controversial, and the Frascati criteria has been accused of being overly inclusive, thus inflating the severity of impairments [19]. Although the rate and degree of neurocognitive decline in HAND is low, ANI often progresses to symptomatic neurocognitive impairment, even with a suppressed viral load [20]. Thus, the importance of ANI in a clinical setting should not be underestimated. There have also been criticisms that diagnoses based on self-reported measures of functional impairment are imprecise, as they are highly variable and influenced by social, educational and cultural factors [18]. This has posed significant challenges to studies addressing HAND, partly explaining the variable findings and the few replications of associations between risk factors (including genotypes) and HAND manifestations found in previous studies, where different neuropsychological measures were often used for diagnosis [21]. Furthermore, while rapid screening tests for HAD are often less sensitive, NCI for the Frascati criteria can be measured using different batteries of neuropsychological assessments and tests, thus enabling classification according to the Frascati criteria. One of these, which is relatively quick to use, is the international HIV dementia scale (IHDS), shown to be adequately sensitive in developed countries [22]. However, using the same range of neuropsychological tests in low-resource settings such as in sub-Saharan countries like South Africa is often less feasible or valid. This could be either due to the extensive expertise and time needed to administer these test [23] or simply because tests such as the IHDS have been found to be less sensitive and valid in such settings. This may be due to the prevalence of different HIV subtypes in such countries; for example, clade C is the dominant subtype by far in sub-Saharan Africa [5,24,25]. Therefore, additional but specific neuropsychological tests such as the Trail-Making Test and Digit Span Test can be used to assess NCI more appropriately within such settings [23]. In addition, there is a need to develop tests that are appropriate for developing countries which are normed according to those specific populations, rather than norms from studies in developed countries [4,5,24,25,26], such as the central nervous system (CNS) HIV Antiretroviral Therapy Effects Research (CHARTER) studies [7]. It is thus important to note that HAND consists of several, variable phenotypes that can be crudely classified, but not without limitations. 

Although HAD and the final fully-blown subcortical dementia of the pre-cART era have become less frequent, the milder neurocognitive impairments are no less prevalent, with the CHARTER cohort indicating a high prevalence of HAND in over 50% of people living with HIV in developed countries like the USA [7]. The prevalence in developing countries like South Africa is similar, with a wide range from 15 to 60%, depending on the setting [4,5,24,25]. The causes of these cognitive impairments are likely to be multifactorial, and include factors such as cardiovascular and metabolic effects, drug use and depression [27]. This makes current treatment protocols that mainly focus on the HIV infection and control through antiretroviral medication (ARVs) less successful, and it is becoming increasingly important to consider additional contributing factors [2]. As such, while there is biological plausibility in using ARVs with greater CNS penetration to treat HAND, the effectiveness of this approach is still unclear and other types of intervention are also required. 

## 3. Pathogenesis of HAND

The pathogenesis of HAND itself is complex and multidimensional, with current understanding still largely incomplete, although intensive investigations have uncovered several mechanisms involved. A detailed analysis of the pathogenic processes in HAND is beyond the scope of this review and described thoroughly elsewhere [17,28,29]. Two main processes are thought to cause neuronal injury that underlies HAND pathogenesis: direct neurotoxicity by HIV and/or its viral proteins, and, more importantly, indirect neuronal damage through neuroinflammation, the so-called ‘bystander effect’ [17,28,29,30]. Another key pathogenic component relates to resident microglia and astrocytes, in addition to some T cells, that act as latent HIV reservoirs despite cART. The importance of such reservoirs was highlighted when six patients had profound reductions in HIV levels and infection following allogeneic hematopoietic stem cell transplantation, although the exact mechanisms are as yet unknown [31]. HIV enters the CNS early in infection, primarily through infected lymphocytes and monocytes that cross the blood-brain barrier (BBB). Migration across the BBB and CNS tropism may be affected by HIV subtypes, resulting in differential susceptibility to HAND. HIV-1 subtype (or clade) C, for example, is predominant in India and sub-Saharan Africa, and may be associated with decreased CNS severity [32]. Subtype B, on the other hand, is more dominant in the USA and Europe, and was associated with higher pre-cART incidence levels [33]. Migration through the BBB by peripheral mononuclear cells in the blood is increased by astrocytes and microglia releasing chemokines—cytokines that induce chemotaxis, such as macrophage inflammatory protein-1 (MIP-1, or CCL3/CCL4) and monocyte chemoattractant protein (MCP-1, or CCL2) [34]. In the CNS, HIV productively infects macrophages and microglia, and, to some extent astrocytes, although they do not support viral replication [35]. Direct HIV infection of neurons does not occur especially with cART and, therefore, does not explain the cause of neuronal damage and/or apoptosis associated with neurocognitive impairment (NCI) [3,17]. Neurotoxic viral proteins, however, are shed by the virus and released by infected cells (macrophages, microglia, and, to a lesser degree, astrocytes), and can still cause direct neuronal injury [36,37,38,39]. These include viral surface glycoprotein (gp120), transactivator of transcription (Tat) and viral protein R (Vpr). Importantly, these viral proteins are also indirectly neurotoxic, contributing to neuroinflammation (the ‘bystander effect’) through activation of macrophages, microglia and astrocytes by binding to the α-chemokine receptor, C-X-C chemokine receptor (CXCR4) and C–C chemokine receptor 5 [17,28,29,30,39,40,41]. The most intensely studied and perhaps most important viral protein is glycoprotein120 (gp120), a neurotoxic surface protein that enables HIV entry into CD4 T cells, or T helper cells. It has been particularly implicated in the neuropathogenesis of HIV/AIDS, mostly in the presence of glial cells [29,36,41]. Gp120 can also cause excitotoxicity by binding to *N*-methyl-d-aspartate (NMDA)-coupled ion channel receptors (NMDARs) on neurons, resulting in excessive calcium influx, while Tat phosphorylates NMDARs which potentiates glutamate excitotoxicity [38]. Both Tat and Vpr have also been implicated in neuronal damage, with considerable neuronal apoptosis associated particularly with Tat [37,38,39]. Importantly, most subtype C viruses have a mutation in the gene encoding the Tat protein resulting in a Cys31Ser polymorphism which disrupts the dicysteine motif making the protein homologous to β-chemokines [42,43]. This is possibly responsible for decreased neurotoxicity and monocyte chemotaxis, when compared to subtype B viruses [44]. This may be the factor reducing HAND severity in Indian populations [45]. In fact, NMDA-receptor activation by the mutated form of the Tat protein leads to reduced human neuronal cell death in clade C compared to clade B [43,46], data that correlate to elevated neurobehavioral deficits in clade C than clade B-infected mice [44]. This suggests that neurotoxicity may be clade-specific [43,47]. However, direct neurotoxicity by the virus is an infrequent occurrence in HAND pathogenesis, with neuroinflammation playing a far more significant role [17,29]. 

Neuroinflammatory processes in HAND are regulated by perivascular macrophages, microglia and astrocytes [29], which release neurotoxic substances and inflammatory cytokines upon immune activation or viral infection. This leads to impaired neuronal function, injury, and, to some extent, apoptosis [28]. Both infected and activated macrophages and microglia release inflammatory cytokines with direct neurotoxicity, such as tumour necrosis factor-alpha (TNF-α) [48,49], and indirect neurotoxicity, such as interleukin-1beta (IL-1β). Moreover, chemokines are also released by macrophages and microglia, as well as astrocytes, and play an important role in regulating neuronal injury. While α-chemokines (or CXC chemokines) can be neurotoxic [such as stromal-derived factor (SDF)-1)], C–C-chemokines, particularly MIP-1 and RANTES, may have neuroprotective effects and protect against HIV/gp120 binding and neurotoxic processes [29,36,50,51]. Furthermore, infected/activated macrophages and microglia release neurotoxic substances, including ATP, arachidonate, and the excitatory amino acids, glutamate, quinolinate, and cysteine, while astrocytes also release glutamate and nitric oxide radicals [28,52]. Ultimately, this leads to disturbed bioenergetic homeostasis, neuronal injury and dendritic/synaptic pruning through both neuroinflammation and direct neurotoxicity [17]. The release of more inflammatory cytokines, particularly TNF-α, IL-1β, and SDF-1, further activate other macrophages and microglia, inducing the production of TNF-α [28]. Furthermore, gp120 and inflammatory cytokines TNF-α, IL-1β and interferon-gamma (IFN-γ) stimulate activation and proliferation of astrocytes (astrocytosis). Activated astrocytes release nitric oxide that further damages neurons directly through lipid peroxidation [50,52]. Both arachidonic acid and TNF-α released from infected and activated brain macrophages/microglia increase astrocytic glutamate release and impair its uptake and clearance [28,50]. Subsequently, excessive increases in the concentration of the neurotransmitter glutamate produce excitotoxicity [28]. Excitotoxicity occurs when excess glutamate activates neuronal NMDARs, resulting in abnormally high calcium influx, and further free radical (NO) formation, mitochondrial injury and production of reactive oxygen species (ROS) concomitant with lipid peroxidation and caspase activation that damage neurons [17,28,48]. Thus, HAND pathogenesis primarily involves the response of non-neuronal cells (perivascular macrophages, microglia and astrocytes) to HIV infection and specific viral proteins that results in a neurotoxic process. Although higher HIV levels in cerebrospinal fluid (CSF) are associated with HAND, they do not correlate well with the degree of cognitive impairment [6]. This points to the importance of host immune response and host factors in the pathophysiology of HAND, rather than only the direct neurotoxic effects of HIV [41]. Importantly, while subtype C is also predominant in sub-Saharan Africa, levels of HAD are much greater compared to India, and more similar to regions where subtype B predominates [45,53]. This is particularly due to HIV-1 subtype C Tat proteins having an intact dicysteine motif in sub-Saharan variants, unlike the south-east Asian subtype C variants which developed later on. The intact dicysteine motif in the sub-Saharan subtype C Tat protein, being more homologous to β-chemokines (like the subtype B Tat protein), induces a greater rate of monocyte chemotaxis and release of inflammatory cytokines and chemokines (such as CCL2, IL-4 and IL-10) from astrocytes and microglia than clade B Tat protein [44,46,53,54]. This points to the importance of geographical location, as well as viral genotypes and phenotypes in the pathogenesis of and risk factors for HAND.

## 4. Risk Factors for HAND

Several risk factors for HAND have been described, including lower levels of education, early immunosuppression, increasing age, and increased plasma concentration of TNF-α, MCP-1, and nadir CD4 T cell levels, in particular [6,17]. Moreover, cardiovascular factors such as blood pressure and hyperlipidaemia were found to worsen neurocognitive function in HAND [17]. Since many of these factors are controlled by the genetic make-up of the host, there is ongoing research into which genes may be associated with development and/or progression of HAND. Thus far, two genome-wide association studies (GWAS) have been conducted [55,56] and several candidate gene studies have investigated genes implicated in HAND and/or NCIs in HIV infected individuals (for an older, but thorough review, see Kellianpur and Levine (2014) and Levine, Parvos and Horvath (2014) [57,58]). Neither GWAS found any genes associated with HAD or milder neurocognitive impairments, as assessed by neuropsychological tests or global deficit score (GDS), that reached the genome-wide significance threshold. However, the latter GWAS by Jia et al. (2017), looking at GDS, found some biologically plausible gene variants previously associated with Alzheimer’s disease (*SH3RF3* and *CSMD1*), as well as four single nucleotide polymorphisms (SNPs) at the T-cell receptor alpha locus, involved in neurogenesis, which did approach significance [55]. Both studies were, however, limited by sample size, indicating a need for further research to support and validate previous findings. In the present paper, we aim to provide a brief overview of some of the genes and the related mechanisms that have been found to be associated with HAND development and progression, and specifically those found in candidate gene studies.

## 5. Genes Involved in Neurotransmitter Systems

In the brain, the neurotransmitters dopamine and serotonin are associated with the control of executive functions, memory and attention. Several genetic polymorphisms of those neurotransmitters are associated with cognitive functions [59]. Although these were found to have small effects, they may have more significant impact with pre-existing cognitive deficits, such as in HAND [60]. In view of this, several studies have investigated the effect of genetic polymorphisms related to dopamine and serotonin neurotransmission on cognitive functions in HAND [60,61,62]. 

### 5.1. Genes Related to Serotonin Neurotransmission

Two SNPs of serotonin-related genes have been associated with NCI in HIV-positive(+) adults, particularly in African American males [61,63]. A homozygous TT genotype in the SNP rs4570625 was associated with poorer executive functioning in HIV+ individuals, most of whom were with ANI, while a small proportion had MNI (HAD was not found in any participants), as assessed by Frascati criteria [61]. This SNP is located in the transcriptional region for the gene encoding tryptophan hydroxylase 2 (*TPH2*), the rate-limiting enzyme in serotonin biosynthesis, and affects the transcription rate of *TPH2* [61]. The same study further found that the polymorphism *GALM* rs6741892 of the galactose mutarotase gene (*GALM*), which indirectly increases local serotonin release, was linked to impaired memory in the same participants [61]. The *SLC6A4* 5-HTTLPR polymorphism, associated with serotonin transport, had no significant effect. These results were in agreement with earlier findings of associations between the same SNPs and poorer executive function and memory [63], with some limitations due to the cross-sectional design and lack of a healthy control group. 

### 5.2. Genes Related to Dopamine Neurotransmission

Other studies have looked at the effect of genetic polymorphisms related to the neurotransmitter dopamine, which is involved in executive function, working memory, and drugs-of-addiction and reward pathways in the CNS [60,64]. Dopamine is largely implicated in the fronto-striatal system, which consists of connections between the midbrain and prefrontal cortex, areas important for various cognitive functions. Studies have found that the fronto-striatal system is particularly affected in HAND and HIVE, demonstrating both structural and metabolic abnormalities and pathology (for review, see Hauser and Knapp, 2014) [65]. Chronic HIV infection results in depleted levels of dopamine and a reduced quantity of dopamine receptors in the basal ganglia, with dopaminergic neurons being particular vulnerable to injury by Tat and gp120 [65]. In the context of HAND, the dopamine-related genes that have been studied include genes for: catechol-*O*-methyltransferase (*COMT*), an enzyme involved in dopamine metabolism; the dopamine transporter (*DAT*), the channel for the re-uptake of dopamine from the synaptic cleft; brain-derived neurotrophic factor (BDNF) which is involved in regulating dopaminergic and serotonergic activity; and dopamine beta-hydroxylase. Those genotypes have been found to affect cognitive functioning in HIV-negative(−) populations, but their role in HAND is still unclear due to the limited number of reported studies [60]. Nonetheless, the genes encoding COMT and dopamine receptors do seem to be associated with various measures of NCIs in HAND populations [64,66,67,68]. Regarding the *COMT* gene locus (rs4680), it was found that the Met/Met genotype (*val158met*) was associated with improved performance in various executive functioning tests in HIV-infected individuals with mild cognitive impairment, but not in those concurrently using methamphetamine [66]. This is due to this variant resulting in reduced COMT activity in metabolising dopamine, thus increasing levels of dopamine [66]. SNPs in the dopamine receptor gene *DRD2* (rs6277), which affect D2 receptor density in the striatum, is associated with somewhat impaired cognitive flexibility and executive function in adults with HIV [64]. Variations in the *DRD4* gene (48 base pair-variable number tandem repeats) were also linked with impaired executive function, which affect both D2 receptor density and binding affinity [64]. Similarly, *DRD1* and *DRD2* gene polymorphisms have main effects with substance use on neuropsychological performance in various cognitive domains in HIV+ individuals [68], with opposite effects in those who used substances compared to those who did not. Furthermore, a genetic polymorphism of *DRD3* (rs6280TC)—resulting in a serine to glycine amino acid substitution and subsequent increase in dopamine binding affinity of the D2 receptor DRD3, also found in macrophages—was linked to considerable cognitive impairment in HIV-infected methamphetamine (meth) users [67]. That dopamine-related gene variants influence cognitive impairment in HAND also has biological plausibility considering a different mechanism: monocyte infection and chemotaxis is increased by extracellular dopamine concentrations [69]. The gene variants mentioned cause changes of extracellular dopamine and affect the binding affinity and density of DA receptors expressed in monocytes, such as DRD1, DRD5, and DRD4 [69]. The findings linking dopamine-related genes to cognitive impairment, however, may not be specific to HAND populations as they have not been adequately replicated in the context of HAND, and because similar trends have also been observed in HIV negative populations. In fact, the two GWAS studies [55,56] did not replicate those relationships with the *COMT*, *DRD1*, or *DRD2* gene polymorphisms. Importantly, a longitudinal study [60] extending over 10 years did not find any effects of the genetic polymorphisms of *COMT val158met*, *BDNF val66met*, and the *DAT* genes on neurocognitive functioning (as assessed by comprehensive neuropsychological exams) in HIV+ populations, but did not include people with HIV dementia. Another extensive candidate gene study, which did assess HIV dementia scores, found no associations either [62]. 

## 6. Genes Affecting Integrity of Mitochondrial and Nuclear DNA in HAND

HAND pathogenesis, to a large degree, involves the production of ROS and oxidative damage which affect both nuclear and mitochondrial DNA structure and function, ultimately leading to neuronal apoptosis [17,70]. One important pathogenic mechanism in HAND includes Tat-induced double strand breaks in DNA which is lethal to cells if left unrepaired [71] (for review, see [71] for HIV-induced mitochondrial toxicity and [72] for mitochondrial DNA (mtDNA) haplogroups related to HIV infection and treatment). Only a few studies, however, have investigated both nuclear and mitochondrial oxidative DNA damage in neurocognition in HIV, and its role in neuronal apoptosis and HAND pathogenesis [73,74,75]. Higher levels of mtDNA damage are associated with poorer general neurocognitive function in HIV+ patients [71]. Moreover, a frequently found mtDNA deletion mutation (known as the “common deletion”) was associated with NCIin HIV+ patients using methamphetamine, as assessed by a continuous GDS (with a GDS > 0.5 being the criteria for NCI) [70]. This mutation, consisting of a 4977 base pair deletion affecting genes involved in the respiratory chain, causes mitochondrial dysfunction and is more common in brain tissue in neurodegenerative diseases [70]. Furthermore, post-mortem tissues from the prefrontal cortex in HIV− and HIV+ patients with and without non-specific HAND (assessed by the International HIV Dementia Scale) were compared and modification of 8-hydroxydeoxyguanosine (8-oxoG), a marker for oxidative damage, examined [74]. Patients with HAND of any severity had particularly high levels of nuclear and mitochondrial DNA 8-oxoG damage, compared to HIV+ patients without HAND and HIV− controls. Furthermore, mtDNA in the HAND group was found to have a much greater number of non-coding displacement (D) loop mutations. Both of those features indicate that higher levels of ROS contribute to HAND pathogenesis [74]. Interestingly, oxidative stress and neuronal apoptosis induced by gp120 and Tat can be reduced by the delivery of antioxidant enzymes through gene transfer [73]. Another cross-sectional study, which looked at GDS results and Frascati criteria for HAND diagnosis, found that mtDNA haplogroup B is associated with less NCIand decreased prevalence of HAND of any severity in individuals with Hispanic ancestry [76]. No associations of mtDNA variations with HAND were found in individuals of European or African ancestry. Thus, the ‘common deletion’ may be a risk factor of HAND development, with mtDNA haplogroup variation having an ancestry-specific influence on neurocognitive impairment in HAND. 

## 7. Telomere Length and Cell Age

Telomerase activity is closely related to oxidative damage. This enzyme extends the length of telomeres, structures of repetitive DNA sequences at the end of chromosomes that protect against the shortening of DNA during cell division. Telomere length also gives an indication of biological age, since they shorten with each cell division cycle depending on the level of telomerase activity. Telomerase activity is impaired by oxidative stress [57], and telomere length decreases with chronic psychological stress [77]. In the context of HAND, there was a positive correlation between longer telomere length in leukocytes and learning performance (as part of the International Neuropsychological Test Battery developed by the HIV Neurobehavioural Research Centre) in a group of HIV+ women in South Africa (HAND severity was not indicated) [77]. Shorter leukocyte telomere length may also indicate immune senescence, suggesting accelerated cellular aging in HIV disease [78]. However, these findings were not replicated; a different study found no associations between leukocyte telomere length and overall neurocognition (assessed by a comprehensive neuropsychological battery, with mild to moderate NCI) [79]. Comparing biological age to chronological age by measuring the levels of epigenetic DNA methylation can also indicate whether accelerated aging is a feature of HAND. In fact, epigenetic age is increased in HAND, but has not been associated with HAND severity [80]. Moreover, the protein HIV-1 Tat has been found to upregulate the expression of histone deacetylase-2 (HDAC2) in human neurons [81], resulting in a decreased number of acetyl groups and subsequent chromatin condensation with reduced transcription.

## 8. Genes Related to Cytokines and Associated Receptors of the Immune System

### 8.1. Gene Variants of CCR2 and Associated Ligand CCL2 (MCP-1) 

MCP-1 has been associated with HAND more consistently than most other gene products. Increased levels and expression of MCP-1 were found in the CSF in HIV-infected patients with increased GDS (indicating greater cognitive impairment) [82] and in astrocytes in AIDS dementia [40]. MCP-1 plays an important role in neuroinflammation in HAND, as it draws immune cells, monocytes in particular, to the CNS across the BBB [83]. However, it has also been shown to protect neurons from apoptosis induced by NMDA or HIV Tat. Several studies have looked at genetic polymorphisms in the *MCP-1* gene and Prep 1, a transcription factor binding to the *MCP-1* promoter region, as well as the MCP-1 target receptor CCR2 [40,55,56,84,85,86,87,88,89,90,91]. One SNP in the *MCP-1* gene (rs1024611), resulting in the MCP-1-2578G allele which is more easily transcribed, increases CSF MCP-1 concentrations [86] and possibly monocyte infiltration [82]. Importantly, this allele has been associated with a highly increased risk of AIDS dementia [84] and greater GDS along with other CSF pro-inflammatory markers [82]. The longitudinal study by Levine et al. (2014) also found a lack of improvement in working memory compared to HIV+ participants (not including HAD patients) with the allele, albeit a small effect [60]. The most recent GWAS study showed that this same *MCP-1* G allele was linked to a decreased risk of NCI as indicated by GDS, but only with nominal significance [55]. Other studies, however, did not find these associations in adults with AIDS dementia or neurocognitive impairment in HIV-positive children [87,88,89], nor did the other GWAS [56]. These inconsistent findings may be due to variations in the *PREP-1* gene polymorphisms that affect transcription of MCP-1 [89]. Indeed, the heterozygous genotype for SNP rs2839619 in the *PREP-1* gene was much less common in HAD cases compared to controls, suggesting a protective effect [89]. Several studies have also looked at polymorphisms of the C–C chemokine receptor type 2 (CCR-2), which is the natural ligand for MCP-1 and a minor HIV-1 co-receptor. Only a single study found associations between *CCR-2* gene polymorphisms and HIV-associated neuropsychological impairment, where the *CCR2-64I* allele was associated with a greater rate of progression to NCI over time in adults [87]; several other studies found no effects with NCI or AIDS dementia in HIV+ adults [91,92], or impairment in various cognitive domains in children [85] with HIV.

### 8.2. Gene Variants of CCR5 and Associated Ligand CCL3 (MIP-1α)

MIP-1α (or CCL3) is a C–C chemokine secreted by microglia and astrocytes, and binds to its natural ligand CCR5, one of the most important HIV co-receptors [41]. Due to its competitive binding and protection from gp120-induced apoptosis in neurons, MIP-1α, and MIP-1β (CCL4), have shown neuroprotective roles in vitro [48]. The longitudinal study by Levine et al. [60] found that one SNP resulting in a homozygous or heterozygous A allele in the MIP-1α gene (*rs1719134*), instead of a GG allele, was associated with declining ability in learning and memory in adults with HIV, but not HAD. Similarly, an earlier study found that the AA allele at another SNP in the MIP1-α gene (*rs1130371*) increased the risk of HAD two-fold [88]; another study demonstrated no differences in HAD [89]. Other studies have looked at copy number variants (CNV) in the MIP-1α gene, particularly *CCL3L1*. An increased *CCL3L1* copy number may hinder HIV binding and infection of cells by increasing MIP-1α production, outcompeting HIV for CCR5. While a below average *CCL3L1* copy number was associated with higher susceptibility to HIV/AIDS progression [34,93], one study found no differences in *CCL3L1* CNV between NCI, mild NCI, or HAD, while another found no differences between no NCI and NCI (as determined by GDS > 0.5) in a Chinese cohort [90,92]. MIP-1α may thus play a dual role in HAND: while it binds to neurons and prevents neurotoxicity from HIV/gp120, it is also a potent chemoattractant, and variations in its genotype (particularly SNP *rs1719134*), may increase monocyte CNS entry, exacerbating neuroinflammation [21]. 

The CCR5 receptor is a crucial co-receptor for HIV entry into monocytes and microglia, and mutated CCR5 affected HIV disease progression and neurocognitive impairment in the pre-CART era [94,95]. Importantly, a meta-analysis found that *CCR5* locus variation, together with the human leukocyte antigen (HLA) locus explained the large majority of variation in set-point viremia; increased HIV disease progression was associated with variants (*CCR5P1* haplotype) in the *CCR5*-promoter region [96]. The CCR5 Δ32 allele (*rs333*) arises from a deletion of 32 base pairs within the *CCR5* gene, and in loss of *CCR5* receptor expression and function [97]. This mutation protects against infection in those homozygous for the allele, while those heterozygous for the gene also had delayed disease progression [97]. Indeed, the only known patient in whom HIV-1 was completely eradicated (the “Berlin patient”) had received an allogeneic hematopoietic stem cell transplant, with the donor being homozygous for the *CCR5∆32* allele. This polymorphism likely prevented HIV infection of the graft, thus allowing eradication of the virus [98,99]. Examination of neurocognitive impairment in children with HIV revealed that this mutation, but not other SNPs of the *CCR5* gene, slowed disease progression and decreased neurocognitive impairment [85]. The *CCR5 Δ32* allele is also found at a lower prevalence in people with AIDS dementia [91,100]. This finding, however, may be specific to the pre-CART era and different viral loads, since later studies with patients on cART did not find this association [87,89,92]. In fact, Bol et al. (2012) noted that this effect occurred only in those developing AIDS prior to 1991, before cART was introduced [89]. It may be helpful to investigate how *CCR5* mutations correlate with specific features of neurocognitive function and the degree of impairment, rather than simply the diagnosis of HAD. Indeed, a 24-week treatment with a CCR5 antagonist (maraviroc) produced improvements in neurocognitive function in HIV+ adults on cART [101]. This highlights the potential role of *CCR5* and its genotypes in treatment of HAND. 

### 8.3. Gene Variants of TNF-α

TNF-α is a pro-inflammatory neurotoxic cytokine that plays a role in HAND by increasing BBB permeability and activating NMDA receptors, resulting in excessive, neurotoxic calcium increase in neurons [49]. The TNF-α-308 allele (rs1800269) is associated with increased TNF-α production, and is more common in patients with HAD, compared to HIV+ or HIV− controls [102,103]. Other studies, however, found no association between this TNF-α genotype and HAD [88,89] or any effect of TNF genotype on HIVE [104,105]. Increased gene expression related to the TNF-α signalling pathway [106] in HIV infected adults, and TNF receptors [107] in AIDS dementia, have been observed, but this is controversial as other studies did not find an association between HAND of any severity and neurocognition [108,109]. Interferon-α (IFN-α) is another inflammatory cytokine that may be more important due to its role in virus-specific immune responses and activation of astrocytes, and has been positively associated with HIVE [106]. 

### 8.4. Gene Variants of the Chemokine Ligand CXCL12 (SDF-1)

CXCL12, or SDF-1, is an α chemokine (CXC chemokine group) that binds to CXCR4, an important chemokine receptor expressed on neurons, microglia and astrocytes [28]. It is also the main co-receptor for HIV through which T cells are infected [50]. SDF-1 prevents HIV binding to CXCR4 by competing for it and down-regulates *CXCR4* expression [110], but has been implicated in direct neuronal toxicity. It is increasingly expressed in HIVE, and has been implicated in neuronal apoptosis [36,50] and astrocytes exposed to HIV-1 [107]. A gene variant of *SDF1*, consisting of a SNP with a G to A substitution in the 3′ untranslated region of the *SDF-1* gene (*SDF1-3′*-A/A polymorphism; *rs1801157*) has been implicated in HIV disease progression [111]. Compared to the *SDF1*-3′-G/G wildtype, the SDF1-3′-A/A genotype increases production of SDF1 and reduces AIDS progression [111]. However, several other studies found no such association [91,112,113]. Still yet, these studies did not specifically analyze associations with HAND, considering only disease progression in general. In the context of HAND, children homozygous for the *SDF1*-3′-A/A genotype experienced a much higher rate of disease progression and had a greater prevalence of neurocognitive impairment compared to those with the *SDF1*-3′-G/G (wild type) genotype [85]. This replicated earlier findings which linked greater HIV disease progression to the *SDF-13′*-A allele, in children [114]. This, however, may be a finding specific in children, as other studies did not echo any associations with the *SDF1-3′*-A/A genotype and neurocognitive functioning in adults [60,88,92,115]. Nevertheless, one study did indicate that *SDF1* gene expression in astrocytes was up-regulated in patients and possibly implicated in HAND [107].

### 8.5. Genetic Variation of Interleukins in HAND

Interleukins are a large group of cytokines that are secreted by numerous immune cells and are crucial to immune system regulation. Neuroinflammation in HAND is also regulated by several interleukins, such as IL-6 and IL-10 which have been linked to BBB crossing by monocytes, and IL-1β which is a neurotoxic inflammatory cytokine released by activated microglia [41]. With regard to HIV, an *IL-2* gene variant reduces HIV infection risk [116], and a SNP in the *IL-1* gene differed in those with rapid disease progression from those with slow progression [117]. Furthermore, SNPs in the gene coding for IL-1α predicted HIV-1 replication and levels of viraemia [118]. A number of studies investigated associations between SNPs in interleukin genes and neurocognitive function in HIV, but none reported any significant findings. Regarding IL-1β gene variants, neither the IL1-1β*2 allele, nor the interleukin-1 receptor antagonist (*ILIRN*)*2 allele (which regulates IL-1β release), had an effect in HIVE [105]. Furthermore, allele frequencies of IL-1α −889 (*rs3783525*), IL-1β +3953, and IL-12 were not different in people with HAD compared to controls [103], and neither were alleles of *IL-1α* (rs17561) or *IL-10* (rs1800872) [88]. Lastly, a polymorphism in *IL-4* (IL4-589-C/T) was not associated with neurocognitive function in individuals with HIV [92]. Thus, it is unlikely that genetic variants of interleukins specifically have a significant effect on neurocognitive functioning in HAND, even though these cytokines play an important role in HAND pathogenesis, as indicated by gene expression studies [107]. 

## 9. Mannose Binding Lectin (MBL-2)

Variations of the *MBL-2* allele have been associated with HIV infection and AIDS progression [119], likely due to the role of MBL-2 in macrophage-related innate and adaptive immunity. For example, one study found that in children, homozygous for the B, C and D alleles (*rs1800450*, *rs1800451* and *rs5030737*, respectively), experienced greater cognitive decline [120]. Those findings were supported by a study of a cohort of Chinese adults, where the same genotypes were associated with mild NCI and HAD [92]. Levine et al. (2014), however, did not replicate these findings in HIV positive adults with NCI (excluding HAD), and only the HIV-control group showed an association between the ‘O’ *MBL-2* genotype and NCI. This suggested that this relationship was not associated with HIV comorbidity [60]. *MBL-2* expression is increased in neurons in HIVE [121], but this relationship has not been correlated with neurocognitive functioning. Although associations of SNPs of MBL-2 were specifically evaluated in each GWAS, no significant findings were reported [55,56]. It is therefore unlikely that *MBL-2* has a specific role in neurocognitive impairment in the context of HIV, although earlier findings that did indicate associations warrant further research. 

## 10. Human Leukocyte Antigen Class I and II (HLA) Genes

HLA is the protein machinery that presents antigens to immune cells on the cell surface. There are two classes of HLA, class I and class II, both encoded for by highly polymorphic genes. HLA class I genes (*HLA-A*, *HLA-B*, *HLA-C*) code for cell surface proteins that ‘present’ antigens produced by the host cell itself (which can include viral proteins if a cell is infected). HLA class II genes (*HLA-DR*, *HLA-DP*, *HLA-DQ*) code for molecules that present viral antigens engulfed by immune cells [122]. HLA I is important in viral immunity and implicated in HIV disease progression, as infected cells present viral antigens via HLA I to cytotoxic T lymphocytes (CTL), also known as CD8 T cells, in order to enable CTLs to recognize and kill infected cells [122]. For an effective CD8 T cell response, however, T helper cells (CD4 T cells) are required, which are activated by HLA II [123]. HLA alleles, particularly HLA class I alleles, affect HIV disease progression (for review, see Naranbhai and Carrington, 2017 [122]). Some of the same alleles (HLA-B*27, -B*57, -B*58:01) have also been associated with reduced HIV-associated neurocognitive functioning in adults (with and without AIDS dementia) [123] and children [124] with HIV. A study in China found that increased NCI (determined by GDS), as well as a greater rate of neurocognitive decline (over 12 months), occurred in adults carrying the HLA-DR*04 allele (an HLA class II allele which codes for a low CD4 T cell response) [123]. Furthermore, several HLA class I alleles (specifically, HLA-A*03, -A*33, HLA-B*27, -B*57 and -B*58:01) had protective effects, with less neurocognitive impairment at baseline and lower rates of decline. Those protective HLA class I alleles are associated with CD8 T cell responses to more conserved regions of HIV [123]. The HLA-DR*04 allele reduced the protective effect of those HLA class I alleles. This highlights the importance of the additional role of CD4 T cells in the control of HIV by CD8 T cells. Taken together, these findings support an earlier study of an American cohort, where the HLA-DR*04 allele was associated with greater risk of HAND [123]. With regard to children, the HLA-B*27 genotype, a gene variant of HLA class I, was also found to protect against neurocognitive impairment and the delay onset of CNS impairment [124]. In addition, further protective effects were associated with the *Cw-2* allele and HLA class II *DQB1-2* allele [124]. In contrast, the HLA-A*24 genotype was associated with greater CNS disease progression. Both these studies correlate well with previous findings relating HLA alleles to HIV disease progression [122]. The expression of genes encoding HLA class 1 *HLA-A*, *HLA-B*, *HLA-C*, *HLA-G*, *HLA-F* are upregulated in response to interferon type I and II in HIVE [106]. Similarly, *HLA-E* was highly upregulated in patients with HAND (severity not specified) and HIVE [108], as was *HLA-B* [125]. In a more recent study, the HLA-DR genotype did not correlate significantly with neurocognitive functions (assessed as a global clinical rating; GCR) or histopathological markers in HIV-infected adults [21]. Research investigating the role of HLA genes have possibly been limited possibly due to the large sample sizes that are required for HLA genotyping. Given the clear role of HLA alleles on HIV disease progression and potential associations with cognitive impairment, more studies are required to investigate the impact of HLA gene variants in the context of HAND [122]. 

## 11. Matrix Metalloproteinase (MMP) Genes

Matrix metalloproteinases (MMPs) are enzymes involved in remodelling of the extracellular matrix, and, together with the associated natural tissue inhibitors of MMPs (TIMPs), play a crucial role in chronic inflammation and HAND pathogenesis [73]. Increased expression and activity of MMPs and imbalances with TIMPs, cause injury and subsequent increase in BBB permeability by breaking down the tight junctions of endothelial cells and the extracellular matrix [126]. This results in increased migration of infected and uninfected immune cells into the CNS and may thus lead to development of HAND. The genetic polymorphisms of some of these have been implicated in HAND pathogenesis, although there are few studies and no replications of findings. For example, a case control study found that the *MMP-21 572 C/T* genotype was associated with increased risk of HAND development, as were polymorphisms of *MMP-1* (-1607 2G/1G) and *MMP-3* (*MMP-3-1612 6A/5A* allele) [127]. Other *MMP-3* alleles (*MMP-3-1612 5A* allele and *MMP-3-1612 5A/5A* allele) presented a lower risk of HAND [128]. A polymorphism of the MMP-2 gene (MMP-2-735 C>T) has been implicated in risk of HAND development and severity, both alone and synergistically with a *MMP-9* variation (*MMP-9-1562 C>T*) [129]. A SNP of the *MMP-7* gene, resulting in *MMP-7-181 A* or G genotypes, was not associated with HAND, even though MMP-7 is upregulated by the Tat protein [130]. Similarly, *MMP-8* gene polymorphisms (−799C/T and +17C/G) were not significantly associated with a risk of HAND [131]. All these case control studies included HAND of any severity, diagnosed by having an International HIV-associated Dementia Score (IHDS) of less than 9.5. Thus far, these findings have not been replicated, and further research is required to support the reported associations between MMP polymorphisms and HAND. Nonetheless, gene expression of various MMPs is upregulated in HAND, and play a significant role in HAND pathogenesis [73].

## 12. Contribution of Host Genes in Alzheimer’s Disease (AD) and HAND

The most common cause of dementia is the degenerative disease AD. Both HAND/HAD and AD share common histopathological, pathophysiological and genetic features. Synaptic damage in particular appears to be common to both disorders. A study in the South African context reported the prevalence of dementia in a low-income rural community to be 8–12% for those aged 60 years or more, and 11% for those above 65 years of age [132]. Therefore, the expected burden of dementia in South Africa is greater than expected. That study did not, however, seek to investigate other risk factors for dementia such as the apoE4 genotype linked to Alzheimer’s disease (AD). AD appears to occur as a consequence of a sustained neuroinflammatory process caused by a plethora of different factors such as trauma, oxidative agents, infection, and oligomers of tau and Aβ [133,134]. Histologically, features of AD include extensive cerebral atrophy coupled to neuronal loss, formation of characteristic neuritic plaques comprising an extracellular core with Aβ amyloid and entangled neuritic processes, appearance of neurofibrillary tangles with hyperphosphorylated tau protein, and synaptic loss [134,135,136]. In approximately 98% of AD cases, the typical form of the disease is a sporadic or a late onset syndrome, mostly affecting patients older than 65 years of age. Of the 695 genes associated with AD, the most important is that encoding the lipid transporter apolipoprotein E (apoE) found on chromosome 19 (*19q13.2*) [137]. ApoE, produced primarily by astrocytes and activated microglia, is also responsible for the modulation of synaptic plasticity as well as facilitation of brain repair pathways in response to injury [138,139,140]. Among the three apoE alleles (*apoE2*, *apoE3*, and *apoE4*), homozygosity for the *apoE4* allele is a strong genetic risk factor in those patients for developing late-onset AD. It should be noted that individuals with the *apoE4* genotype generally experience cognitive decline twice as fast as non-carriers of the *apoE4* allele [141,142]. Compared to *apoE2* and *apoE3*, the *apoE4* allele increases, at a high rate, the conversion of amyloid-β precursor protein (APP) into β-amyloid via a non-canonical mitogen-activated protein (MAP) kinase signaling cascade. This involves phosphorylation of MAP Kinase Kinase 7 (MKK7) by the dual leucine-zipper kinase DLK which then phosphorylates extracellular signal-regulated kinase (ERK)1/2. Phosphorylation of c-fos by ERK1/2 stimulates the transcription factor activator protein 1 (AP-1) inducing APP gene transcription, thus enhancing production and accumulation of Aβ that injures neurons [143].

HIV-infection accelerates and accentuates premature aging of the nervous system by potentiating, at least in part, immunological senescence, measured by the accumulation of CD28^−^/CD57^+^ T cells [78,144] and activation of the monocyte markers CD163 and CXCL10 [145]. The neurocognitive performance of young (≤40 years of age) HIV+ patients was found to be equivalent to those of older (≥50 years of age) seronegative individuals [146,147]. Several studies have reported a positive correlation between HAND of any severity and AIDS dementia and β-amyloid deposition in the CNS in patients with the *apoE4* genotype [148,149,150]. Such reports have provided glimpses into the interaction between *apoE4* and HIV infection [92,147,151,152]. Sections of temporal lobe/hippocampus, entorhinal cortex and frontal lobes from the brains of HIV-1 infected patients obtained at autopsy were immunohistochemically stained with antibodies against Aβ and phosphorylated-tau (p-tau) [149,153]. In contrast to the neuritic Aβ plaques observed in the AD brain, diffusely distributed Aβ plaques and sparsely-scattered neurofibrillary lesions were detected in both HIV cases and non-HIV controls [144]. Those features correlated with aging rather than symptomatic AD. *ApoE4* increases somatodendritic tau accumulation and neuroinflammation in mice expressing the human *apoE4* isoform [154,155,156]. In a gp120-transgenic mouse model of HAND, levels of total and p-tau were elevated in hippocampal dentate gyrus granule cells and CA3 pyramidal cells, which is important given the role of the hippocampus in learning and memory [157]. In postmortem brain samples from ART-treated HIV-infected patients, p-tau levels were increased compared to age-matched non-HIV subjects [158,159,160]. An association between the *apoE4* genotype and tau pathology in HAND is not clear. 

Interestingly, the abundance of cortical Aβ deposition and synaptic loss was associated with HAND of any severity only in those individuals carrying the *apoE4* allele [149], a feature that correlated with poor cognitive function and performance [92,161]. In fact, there are strong associations between *apoE4* and the rate of HIV-1 disease progression (enhanced viral attachment and fusion) [151], and between *apoE4* and HAD in older (≥50 years) HIV-1 infected patients. This is despite the fact that those same patients do not show an increased risk for HAD [147,162]. Moreover, in an 11-patient HIV cohort with and without the apoE4 allele, patients with the *apoE4* genotype were found to exhibit very mild/moderate dementia [152]. The human *apoE4* isoform is also the least effective in Aβ clearance compared to apoE2 or apoE3 [163]. Structurally, the N-terminal domain (amino acid residues 1–191) of apoE contains the receptor-binding region, while the C-terminal domain (residues 206–299) consists of amphipathic α-helices and the high-affinity lipid-binding region (residues 244–272) [164,165]. The apoE4 isoform preferentially binds to triglyceride-rich very low density lipoproteins (VLDL) [166], with evidence indicating that the amphipathic α-helix domain of *apoE4* may be important in facilitating HIV-1 fusion by binding to gp41 [167]. This contrasts with apoE3 which inhibits HIV infection [168]. Importantly, a truncated form of *apoE4* (residues 1–244) has been found in AD brains in which the lipid binding C-terminal domain induces CNS toxicity [169]. HIV+ patients with symptomatic neurocognitive impairment expressing the apoE4 allele also exhibited enhanced glial metabolite myo-inositol:total creatine ratios [170] and reduced neuronal metabolite *N*-acetyl aspartate:total creatine ratios, indicating cellular injury or aging [171]. Persistent glial (astrocytes and microglia) cell activation was detected in the basal ganglia, frontal white matter and parietal cortex [170]. Those individuals also performed poorly in cognitive tests [51,170], suggesting susceptibility to HAND. The introduction of cART has led to dramatic reductions in morbidity and mortality in HIV-infected patients. Even with cART, chronic HIV-infection continues to drive systemic immune activation and an inflammatory response (HIVE) [172,173]. In HIVE, the BBB barrier is disrupted and microglia and astrocytes are persistently activated [30,35,174]. cART drugs also disrupt microglial function, increasing neuronal synthesis of Aβ [175] but reducing clearance of β-amyloid [176]. The net result is progressive worsening of inflammation and brain damage. Therefore, it is likely that a neuroinflammatory response may favor Aβ plaque formation. Neuroinflammation is in fact a critical element in the brain’s response to injury and a common feature when analyzing the progression of HAD and AD. 

Gene expression microarray analysis on HIV-1-infected cultured human neuronal progenitor (hNP1) cells treated with recombinant human *apoE4*, revealed that from the 85% of genes downregulated, 22% of those [including early B-cell factor 3 (EBF3), myelin transcription factor 1, forkhead box C1, neurogenin 2, basic helix-loop-helix family member e22, doublecortin, neurofilament medium polypeptide and internexin neuronal intermediate filament protein alpha], are involved in neurogenesis [177]. Those data, together with earlier evidence implicating *apoE4* as a transcriptional repressor [178] indicates that *apoE4* may be detrimental to neuronal functions, thus suggesting a further mechanism of *apoE4*’s effect on neurocognition in HAND. The hippocampus, being one of the first brain regions to be affected in AD, is particularly vulnerable to injury in this disease [179]. In the dentate gyrus in 10-week-old C57BL/6J mice expressing the human *apoE4* allele, nestin-positive newborn neurons had lower dendritic spine densities associated with fewer dendritic branches [180]. This is significant when considering the potential impact on the learning and memory circuitry and cognitive decline. 

Excitotoxicity is associated with pathogenesis in several neurological injury states including AD and involves excessive stimulation of glutamate receptors with consequent neuronal degeneration. Over activation of glutamate receptors increases nitric oxide and free radical release, and Bax protein activation through elevated levels of intracellular calcium [181]. Tat and gp120 trigger excitotoxicity in the CNS via persistent activation of NMDA receptors causing neuronal apoptosis [37,38,39]. Experimental models further reveal that Tat protein also stimulates Aβ production [182], prevents its degradation by inhibiting the amyloid-degrading enzyme neprilysin [183,184], and promotes the size and numbers of the β-amyloid plaques by potentiating β-site APP cleaving enzyme 1 (BACE1) (or β-secretase) activity [52]. Recently, Stern and others (2018) found increased Aβ oligomer and BACE1 protein expression in the hippocampus and dorsolateral prefrontal cortex of HIV-infected patients diagnosed with HANDs of any severity [185]. The same authors further treated Sprague–Dawley rat cortical neuron cultures with the supernatant of HIV-1-infected human monocyte-derived macrophages and discovered NMDA-receptor-dependent elevation of BACE1 levels. Taken together, those data suggest that BACE1 inhibition may provide a therapeutic option in patients with HAND. Glutathione S-transferase pull-down assays in cultured human fetal neurons showed that apoE4 and Tat are competitive ligands for low-density lipoprotein receptor-related protein 1 (LRP1) [186]. However, according to Liu et al. (2000), Tat uptake/endocytosis into neurons through LRP1, mediated by initial, rapid, and high-affinity binding to the protein heparan sulfate proteoglycan, prevents *apoE4* uptake/clearance [186]. This increases the half-life of *apoE4* in the cerebral circulation, and as *apoE4* microglia are neurotoxic, more so than *apoE3* [187], brain injury may be augmented [186]. 

Diffusion tensor imaging (DTI) is routinely used to detect changes in cerebral white matter integrity [188,189]. A characteristic neuropathological feature in the brain of AD and HIV-infected patients is damage to the corpus callosum; the medial temporal lobe is also vulnerable to injury in individuals with AD and both types of damage correlate to cognitive impairment [190,191,192,193,194,195,196]. The white matter of the body of the corpus callosum is damaged in HIV-patients homozygous for *apoE4*, but not in non-HIV-infected patients negative for *apoE4* [195,197]. Positron emission tomography (PET) scans with the radiotracer ^18F^Florbetaben have been used as a screening tool for the presence of β-amyloid plaques. It is worth noting that several studies found no evidence of amyloid burden in HIV-infected patients with the *apoE4* genotype [198,199,200]. Therefore, it is possible that *apoE4* does not affect HAND. However, as Cysique et al. (2015) suggest, the accumulation of β-amyloid is a gradual process. In that study [200], a possibility is that affected patients with HAND were still relatively young and that the association between the apoE4 allele in HIV+ individuals may thus be age-dependent. This is supported by several studies which found that *apoE4* genotypes had differential effects across age groups (so-called “antagonistic pleiotropy”), with more pathological implications on neurocognitive function in older patients, (see review, Geffin and McCarthy, 2018). CNS HIV infection may not preclude Aβ/β-amyloid deposition [201]. Here, a 71-year-old HIV-infected male on cART, whose *apoE* genotype remains unknown, was diagnosed with mixed HAND and (possibly) AD syndromes [201]. ^18F^Florbetaben was localized to the frontal, temporal, and parietal lobes bilaterally including the posterior cingulate/precuneus, reported by the investigators as consistent with AD [201]. Chang et al. (2013) using magnetic resonance imaging (MRI), found that older (≥50 years) HIV+ and seronegative *apoE4*-carriers had smaller global brain volumes compared to younger (<50 years old) seronegative *apoE4* carriers; when age-matched (<50 years old) to younger HIV-infected non-*apoE4* carriers, younger HIV-infected *apoE4* carriers displayed striatal and general white matter atrophy [202], indicating a role for *apoE4* as a possible risk factor for premature aging. Despite reports that HIV-infected patients carrying the *apoE4* allele have progressively reduced cognitive functions associated with a loss of white matter integrity and cerebral atrophy [203], susceptibility to cognitive impairment conferred by the apoE4 allele in patients with HAND remains controversial. Several studies [105,198,204,205,206,207] did not found any correlation between apoE4 and HIV dementia in infected individuals. These discrepancies may be attributed to an age-dependent effect of the *apoE4* allele.

## 13. Drug Metabolism/Transporter Genes

Understanding the pharmacogenomic outcome of HAND that takes into account inter-individual genetic variations in drug metabolism is key to improving anti-retroviral drug efficacy and safety. Critical to this issue are genes that encode both drug-metabolizing enzymes (such as cytochrome P enzymes), and transporter proteins at the BBB. Polymorphisms in those genes can alter the pharmacokinetic profile of a drug, and classify people into extensive, intermediate or slow metabolizers for a particular drug. The widely-used non-nucleoside reverse transcriptase inhibitor (NNRTI). Efavirenz is often the antiretroviral drug of choice for first-line treatment of HIV [208,209], but also one that produces long-term neuropsychiatric side effects including psychosis, depression, hallucinations or dysphoria [210,211]. Efavirenz plasma levels above 4 μg/mL have been linked to high levels of CNS toxicity [212,213,214]. Efavirenz is oxidized mainly by hepatic cytochrome P450, family 2, sub-family B, polypeptide 6 (CYP2B6) isozyme. The *CYP2B6* gene is highly polymorphic [215] and the *CYP2B6 c.516 G>T* variant appears to strongly predict the pharmacokinetics of Efavirenz [216]. That is, this 516G>T change, specifically in HIV-infected individuals of African-ethnicity, genetically predisposes these patients (“slow Efavirenz metabolizers”) to develop adverse CNS reactions with a frequency of 34–50% [215,217]. This has important consequences for the African continent as it may dramatically increase the burden of psychiatric illness in HIV-infected patients [217,218]. In those individuals, compared to 15–20% of Caucasian and Asian populations [213,219], *CYP2B6* enzyme activity is reduced, impairing the metabolism of Efavirenz and increasing plasma levels of the drug [215,217,220]. Furthermore, the effect of the *CYP2B6 G516T* polymorphism on neurocognitive performance was evaluated in non-HIV-infected individuals who were administered Efavirenz [221]. Elevated plasma levels of Efavirenz did not correlate with worse neurocognitive performance in those patients. Sandkovsky et al. (2017) examined the effect of *CYP2B6* polymorphisms on neurocognitive function in older adults infected with HIV [222]. Higher levels of the Efavirenz metabolite 8-OH-Efavirenz correlated with better neuropsychological functioning while higher Efavirenz levels did not, in contrast to the findings by Johnson et al. (2013) [221]. Furthermore, the *CYP2B6 G516T* genotype was associated with higher Efavirenz levels in plasma, but this did not correlate with neuropsychological functioning. While these findings are perplexing, and the study was limited by its small sample size, it may point to increased metabolism of Efavirenz having a protective effect as its metabolite does not cross the BBB as easily. Whether cognitive function in HAND-positive individuals with the *CYP2B6 G516T* polymorphism is indeed impaired, remains undetermined. In HIV-infected persons, methamphetamine (meth) use and addiction are common and increase the rate of neurocognitive impairment [223,224,225]. Polymorphisms in *CYP2D6* enhance the risk of neurocognitive impairment in a subset of meth users, termed “extensive metabolizers”, and may predispose such individuals who may also be HIV+, to HAND [226]. 

The two major groups of drug transporters for the movement of molecules across the BBB are the mostly primary active ATP-binding cassette (ABC) transporters, and secondary active solute carrier (SLC) transporters. In the CNS, *ABCB1* (*MDR1*) encodes the important efflux transporter protein P-glycoprotein (P-gp) [227]. P-gp is known to associate with more than 1200 drugs [137] and upregulation of *ABCB1* occurs in HAND [215,228,229]. Although the polymorphism 3435C>T in *ABCB1* has been linked to low plasma levels of Efavirenz [230], P-gp, interestingly, has no influence on Efavirenz uptake at the BBB [231]. Johnson et al. (2013), in a cohort of non-HIV-infected adults administered with the P-gp substrate raltegravir, found that the concentrations of this integrase inhibitor in patients’ cerebrospinal fluid were unaffected by the 3435C>T variant [232]. Further studies, with increased statistical power are needed to assess the genomics of drug transporters as related to the pathogenesis and progression of HAND.

## 14. Summary

**Figure 2:**
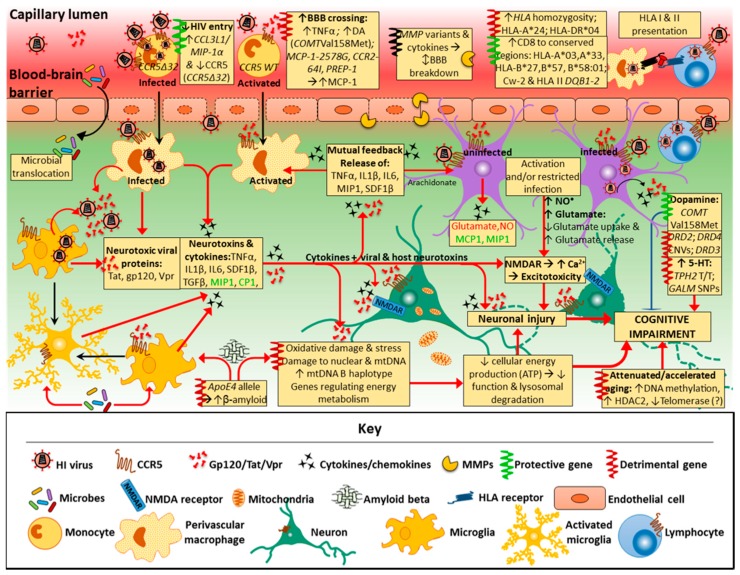
Pathogenic mechanisms and possible genetic influences that lead to development of HIV-associated neurocognitive disorders. HIV and its proteins (gp120, Tat, Vpr) enter the central nervous system (CNS) mainly through infected monocytes and T cells, but also via transcytosis or paracellularly. Infected and uninfected microglia, perivascular macrophages and astrocytes release neurotoxic substances (including viral proteins, if infected), inflammatory cytokines (particularly TNF-α and IL-β), and various chemokines that further activate the same uninfected cells. Neurotoxic substances include gp120 and Tat, quinolinic acid, glutamate, arachidonic acid, and free radicals such as NO, some inflammatory cytokines (TNF-α), and chemokines and cytokines. The resulting neuronal injury is due to functional damage, such as disruption of bioenergetic homeostasis, oxidative stress, damage to synapses and dendrites, and, to a lesser extent, apoptosis. Chemokines may be protective (some C-chemokines, such as MCP-1/CCL2, MIP-1α/CCL3 and MIP-1β/CCL4) or harmful (some CXC or α chemokines, such as SDF-1). Many bind to the chemokine receptor CCR5 expressed by macrophages and many astrocytes and neurons, and importantly, CCR5 also interacts as a coreceptor with gp120, perhaps directly neurotoxic. Increased glutamate is released by neurons leading to excitotoxicity; ATP and cytokines released by macrophages, and decreased glutamate reuptake by astrocytes (as macrophages release arachidonate). This increases intracellular calcium through *N*-methyl-d-aspartate (NMDA)-coupled ion channel receptor (NMDAR)-coupled ion channels activated by excessive glutamate and excitotoxic substances. Finally, this results in neuronal injury through free radicals, oxidative damage, and apoptotic pathways. Potential gene variants may influence pathogenic processes by altering expression of cytokines, chemokines, MMPs, HLA types. Neurotransmitter levels (affecting cognitive processes), β-amyloid deposition, susceptibility to oxidative damage and stress, as well as aging, are further affected by genotypes. Gene variants are marked by small ‘chromosomes’ on the side of text, where green indicates protective and red harmful/neurotoxic effects. (*TNF-α, tumor necrosis factor-alpha; IL-, interleukin; Tat, transactivator of transcription; gp120, glycoprotein120; NO, nitric oxide; MCP-1, monocyte chemoattractant protein; MIP-1, macrophage inflammatory protein-1; SDF-1, stromal-derived factor; NMDA, N-methyl-d-aspartate; MMP, matrix metalloproteinase; HLA, human leukocyte antigen.*)

**Figure 3:**
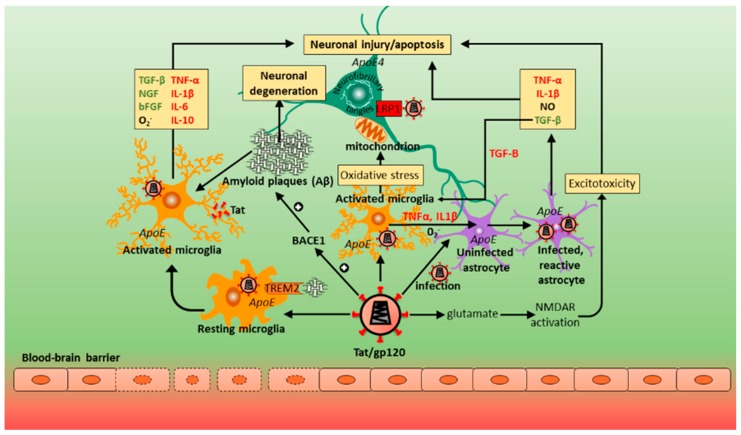
Common pathways in AD and HAND pathogenesis. In AD and HAND, there are several lines of attack that lead to neurodegeneration. β-amyloid aggregates not only have direct cytotoxic effects on neurons, but also indirectly through the generation and progression of a local neuroinflammation via activated and dystrophic resident glial cells in the vicinity of neurons. The latter, reactive gliosis, brings with its secretion of cytokines and chemokines that are released from microglia predominantly, and reactive astrocytes. The interaction between activated microglia and reactive and astrocytes is an important component of HAND pathogenesis. Secreted factors from microglia include members of the complement system (C1q, C3b, C4d, C5b-9), and pro-inflammatory cytokines TNF-α, and IL-1β, IL-6, IL-10, whereas astrocytes release mainly TNF-α and IL-1β. Enhanced secretion of TGF-β from reactive astrocytes favors activation of microglia. Further, TREM2 is highly expressed by microglia and alleviates pro-inflammatory factor-induced AD pathology by mediating phagocytosis of cell debris [233]. Aβ binds to and reduces TREM2 activity; dysregulation of TREM2 signaling contributes to the pathogenesis of both AD and HAND [234]. One interesting aspect is the release of reactive oxygen species (ROS) which can have multiple effects in this inflammatory process. For example, ROS can induce neuronal protein and lipid oxidation, DNA damage, and impair mitochondrial function by disturbing the oxidative respiratory chain, affecting neuronal transport processes and the ubiquitin-proteasome system. ROS, released from Aβ-stimulated microglia can also induce astrocytes to adopt a neurotoxic phenotype. The net result is the worsening of inflammation and brain damage. The HIV proteins Tat and gp120 activate NMDA receptors triggering excessive glutamate release and excitotoxicity. Tat also activates BACE1 promoting the formation of B-amyloid plaques, and enters neurons via LRP1 to prevent apoE4 uptake, amplifying neurotoxicity. Tat can also be synthesized and released in HIV-infected astrocytes; one source of infection is derived from activated microglia. ApoE4 is known to enhance HIV infection [151]. Neurons and microglia also secrete FGF and BDNF in an attempt to promote neuronal survival. Disease progression, however, results in the failure to repair the injured neurons. (*AD, Alzheimer’s Disease; HAND, HIV-associated neurocognitive disorder; TNF-α, tumor necrosis factor-alpha; IL-, interleukin; TGF-β, transforming growth factor beta; TREM2, membrane-bound triggering receptor expressed on myeloid cells 2; ROS, reactive oxygen species; Tat, transactivator of transcription; gp120, glycoprotein120; NMDA, N-methyl-D-aspartate; BACE1, beta-site amyloid-beta precursor protein cleaving enzyme 1; LRP1, low-density lipoprotein receptor-related protein 1; FGF, basic fibroblast growth factor; BDNF, brain-derived neurotrophic factor.*)

HAND consists of a spectrum of neurocognitive disorders and, for affected individuals, is associated with a poorer quality of life and medication adherence, decreased ability to perform complex daily tasks, and has an enhanced prevalence with other age-related diseases. Overall, patients on cART show a decline in the more severe form of HAND, but the frequency of less severe forms of cognitive impairment has increased. The neuropathogenesis is multifactorial, leading to neuronal damage or dysfunction, with CNS inflammation at the core of the disease (Figure 2). HIV crosses the BBB and accesses the CNS via a “Trojan horse” mechanism relatively early (within weeks) following exposure. Here, infected monocytes and lymphocytes enter the brain and activate and infect microglia, astrocytes and oligodendrocytes; the viral particles induce a cascade of events that are toxic to neuronal survival. HIV DNA recovered from brain tissue indicates the presence of latent cellular reservoirs (predominantly microglia/CD16+ macrophages) within the CNS for the virus. In the direct model of HIV-mediated CNS pathogenesis, viral proteins (Tat, gp120, Vpr) released from infected cells cause neuronal death. According to the indirect model, neuronal death is mediated predominantly by inflammation against HIV infection. TNF-α and IL-1β are quite likely the initiators of the inflammatory pathways that cause neuronal apoptosis. In the CNS, HIV-infected microglia and macrophages secrete a large amount of chemokines/cytokines that cause apoptosis, degeneration of the BBB, and glutamate reuptake; thus, chronic inflammation is important in the progression of HAND. However, little is known about potential risk factors of HAND, with some people being more likely to develop HAND than others, indicating the role of host factors in disease pathogenesis. There are multiple host–virus interactions that contribute to HAND, including genetic components (e.g., *apoE4*), metabolic disorders (e.g., insulin resistance), aging (e.g., β-amyloid and p-tau protein) (Figure 3), and vascular disease. Concerning the role of human host genetics in the pathogenesis of HAND, numerous host genes are differentially expressed over the course of HAND, as discussed in this review. Analyzing the contribution of these host genes to neurotropic and/or neurovirulent CNS pathogenesis allows us to understand the neural effects in response to HIV over the course of HAND. The role of host genetic factors in HIV neuropathogenesis and cognitive impairment has been clouded by the critical issue of a lack of reproducibility among various studies. This is particularly due to heterogeneity and ambiguity in measuring disease outcomes (HAND phenotypes and/or measures of cognitive impairment), largely resulting from the variability of HAND presentations, subtypes, and severity. Nonetheless, polymorphisms in a number of host genes that affect common pathogenic pathways implicated in HAND, such as neurotransmitter function, nuclear and mitochondrial DNA integrity, telomere length, cytokine and chemokine activity and chronic inflammation, have differential effects on HAND susceptibility and progression, although effect sizes tended to be small. For example, the *CCL2* (MCP-1) gene variant, *MCP-1−2578G* (*rs1024611*) in HIV+ patients, has been associated with accelerated progression of HIV, accompanied with a 4.5-fold higher risk of developing HAD. Data from other groups, however, have yielded contradictory results, reporting no effect on neurocognitive function in HAND. HIV entry involves an initial interaction between gp120 and the host CD4 receptor, following which gp120 binds to the CCR5 co-receptor on the host cell. HIV infection of macrophages and microglia are mediated by CCR5. A mutation in the CCR5 receptor produces a truncated CCR5 receptor, the CCR5Δ32 variant; heterozygosity for CCR5Δ32 is associated with a reduced risk of impaired cognitive function in patients with HAND. Variants of HLA class I and class II gene have an important influence on the HIV-specific immune response by CD8 and CD4 T cells. Specific polymorphisms of HLA class I have been found to slow HIV disease progression as well as neurocognitive impairment, mainly by encoding a CD8 T cell response that targets more conserved regions of HIV. Some class II gene variants responsible for low CD4 T cell response to HIV may also play a role. More research is needed to establish the effects HLA gene variants have on HAND development, considering the large sample sizes needed for HLA genotyping. Furthermore, BBB disruption occurs through various inflammatory factors, including TNF-α and IL-1β. These two proinflammatory cytokines induce the expression of adhesion molecules allowing for an interaction between microvascular endothelial cells and HIV-infected cells. The host gene variant TNF-308A promotes elevated levels of TNF-α and has been linked to neurotoxicity and progressive neurocognitive severity in the HIV-infected CNS; this has been found particularly in patients with HAD. A polymorphism in the IL-1β 3953T allele is associated with enhanced IL-1β expression. High levels of expression of IL-1β in microglia and macrophages were found in HIV-1-positive patients with HAD. Subsequent studies examining patients with the IL-1β 3953T, however, found no difference between patients with HAD versus HIV+ and HIV− individuals. The same trend was observed from analysing HIV+ patients expressing gene variants of *IL-4* (*IL4-589-C/T*) and *IL-10* (*rs1800872*). Taken together, the current data indicate that gene polymorphisms of interleukins may not be significant contributors to aberrant cognitive functioning in HAND. Data from studies linking the *apoE4* allele to HAND have been inconsistent across cohorts, possibly due to age being an independent risk factor for HAD, and *apoE4* playing a role in older HIV-infected populations, rather than younger adults. Accordingly, HAND pathogenesis has also been associated with accelerated and attenuated aging, linked to mitochondrial DNA mutations, oxidative stress, and related mitochondrial DNA damage (studies on shortened telomere length in HAND are still contradictory). Other genes implicated in the development of HAND are those coding for the various MMPs, but again, their roles have not yet been resolved. Generally, previous studies and reproducibility have been limited in several ways: relatively small sample sizes; lack of variety or inclusivity of certain study populations, with disproportionate focus on Caucasian populations and young adults, with lack of some other ethnic groups and pediatric patients; lack of specificity and clarity regarding disease outcome measures and diagnoses (for example, disease outcomes were often defined by measures of global cognitive impairment or HAND, instead of investigating domain-specific neurocognitive impairments, or HAND subtypes). It would be advisable for future studies to have larger sample sizes with different population groups and more specific outcome measures; the influence on particular cognitive processes and associated impairment and specific HAND subtypes, including ones that are less severe but more common, like ANI. Moreover, since HAND pathogenesis is multifactorial, it is unlikely that single gene variants are responsible or would have large impacts by themselves. Rather, the disease process is influenced by a number of variants that involve several common mechanisms, as shown in this review (such as neurotransmitter systems, immune regulation, aging, and oxidative stress). Thus, studies should investigate common pathways in HAND pathogenesis, combining these with epigenetic, transcriptomic and proteomic investigations as well histopathology and neuroimaging. 

Understanding the influences of the host (and virus) genetic components that contribute to the development of HAND will help clarify the complex process of neuropsychological dysfunction in HIV+ individuals with this disorder. This would aid the development of therapeutically beneficial strategies such as pharmacogenetics procedures promoting neuroprotection against HAND.
